# The importance of the gut microbiome and its signals for a healthy nervous system and the multifaceted mechanisms of neuropsychiatric disorders

**DOI:** 10.3389/fnins.2023.1302957

**Published:** 2024-01-05

**Authors:** Lydia Riehl, Johannes Fürst, Michaela Kress, Nadiia Rykalo

**Affiliations:** Institute of Physiology, Department of Physiology and Medical Physics, Medical University Innsbruck, Innsbruck, Austria

**Keywords:** gut-brain axis, neuropathic pain, migraine mental disorder, schizophrenia, major depressive disorder

## Abstract

Increasing evidence links the gut microbiome and the nervous system in health and disease. This narrative review discusses current views on the interaction between the gut microbiota, the intestinal epithelium, and the brain, and provides an overview of the communication routes and signals of the bidirectional interactions between gut microbiota and the brain, including circulatory, immunological, neuroanatomical, and neuroendocrine pathways. Similarities and differences in healthy gut microbiota in humans and mice exist that are relevant for the translational gap between non-human model systems and patients. There is an increasing spectrum of metabolites and neurotransmitters that are released and/or modulated by the gut microbiota in both homeostatic and pathological conditions. Dysbiotic disruptions occur as consequences of critical illnesses such as cancer, cardiovascular and chronic kidney disease but also neurological, mental, and pain disorders, as well as ischemic and traumatic brain injury. Changes in the gut microbiota (dysbiosis) and a concomitant imbalance in the release of mediators may be cause or consequence of diseases of the central nervous system and are increasingly emerging as critical links to the disruption of healthy physiological function, alterations in nutrition intake, exposure to hypoxic conditions and others, observed in brain disorders. Despite the generally accepted importance of the gut microbiome, the bidirectional communication routes between brain and gut are not fully understood. Elucidating these routes and signaling pathways in more detail offers novel mechanistic insight into the pathophysiology and multifaceted aspects of brain disorders.

## 1 Introduction

Research in recent decades has explored the relationship between the gut and the brain, including inflammatory processes in the intestine, acute and chronic stress, cognitive deficits, and mental disorders (Rhee et al., 2009; Cryan and O’Mahony, 2011; Cryan et al., 2019). Although there is ample literature on bidirectional communication between the gut microbiome, and the brain some findings are ambiguous, sometimes even contradictory. Balancing microbiota in the gut may be a practical and appropriate method for improving mental diseases (Li R. et al., 2022). Some so-called “psychobiotic” probiotic supplementations containing *Bifidobacterium bifidum*, *Bifidobacterium longus*, *Lactobacillus reuteri*, *Lactobacillus acidophilus*, *Lactobacillus plantarum*, *Lactobacillus casei* and *Lactobacillus rhamnosus* for example attenuate anxiety behavior in neuropathic mice (Zhang et al., 2023). Therefore, the complex bidirectional interactions between microbiota and their host are gaining increasing interest in biomedicine.

## 2 Microbiota in the healthy intestine of mice and humans

The earliest evidence of microbial structures in seawater dates back to 3,700–3,800 million years ago (Nutman et al., 2016). The land surface, particularly in South Africa, was colonized around 3,220 million years ago (Homann et al., 2018). Since then, microbiota have developed symbiotic entities with other species whose complex rules and bidirectional interactions are beneficial for both the microbiota and the host. The host’s outer and inner surfaces such as skin or gastrointestinal epithelia are densely inhabited by microorganisms, and the human microbiome in the gut comprises an impressive number and diversity of microorganisms with numerous co-evolutionary associations (Kim et al., 2018). Due to the co-evolution of host and microbiota, symbiotic relationships have evolved, in which the bidirectional interactions between the host and their microflora influence health and disease, for example by impacting host energy, lipid and carbohydrate homeostasis as well as the physiology of organs like kidney, liver, heart or brain (see Sekirov et al., 2010; Cani and Knauf, 2016; Adolph et al., 2018; Stavropoulou et al., 2020; Zheng and Wang, 2021; Shahab and Shahab, 2022; Glorieux et al., 2023; Hsu and Schnabl, 2023; Nesci et al., 2023). Bacterial colonization of the gastrointestinal (GI) tract plays a dominant role in processes of human post-natal development and maturation of the immune, endocrine, and central nervous systems (CNS) (Afzal et al., 2020; Banfi et al., 2021; Hill et al., 2021; Ahmed et al., 2022; Yousefi et al., 2022; Sasso et al., 2023; Van Pee et al., 2023). Important interactions in particular between the microbiota in the gut and the host’s body and even brain as well as disturbed body-to-brain loops have been associated with mental disorders (Bravo et al., 2011; Cryan and Dinan, 2012; Jiang et al., 2017; Heintz-Buschart and Wilmes, 2018; Kim and Shin, 2018; Bassett et al., 2019; Cryan et al., 2019; Mörkl et al., 2020; Megur et al., 2021; Oroojzadeh et al., 2022).

The content of human and mouse gut microbiome show 90 and 89% similarity in phyla and genera (Krych et al., 2013). At first glance, these may seem to indicate a high similarity between the gut microflora of humans and rodents, however key differences, especially in the composition and number of microbes, exist particularly in humans. The ratio of the main phyla *Firmicutes/Bacteroidetes* is higher in humans compared to mice (Guinane et al., 2013; Krych et al., 2013; Alkadhi et al., 2014; Nagpal et al., 2018). Expectedly, the gut microbiota in humans seems to be closer to non-human primates than to mice (Table 1), and the differences in the qualitative composition of the microbiome may be related to the differences in nervous system function limiting the suitability and reproduction of humanized gnotobiotic mouse models, especially if the respective bаcteria have host-specific physiological effects (Park and Im, 2020).

**Table 1:** Common and different microbiota in human and mouse.

Organism	Exclusive genera	Common microbiota	References
Human	*Faecalibacterium*, *Mitsuokellla*, *Megasphera*, *Dialister*, *Asteroleplasma*, *Bifidobacterium*, *Succinivibrio*, *Paraprevotella*, *Lachnospira*, *Phascolarctobacterium*	Is dominated by: *Bacteroides* (27.5%), *Ruminococcaceae* (10.2%), *Clostridiales* (9.7%)	Nagpal et al. (2018) and Park and Im (2020)
Mouse	*Oscillospira*, *S24-7*, *Mucispirillum*	Is predominated by: *S24-7* (44.7%), *Clostridiales* (25.3%)	Nagpal et al. (2018) and Park and Im (2020)
Human and mouse		*Clostridiales*, *Bacteroides*, *Rikenellacae*, *Lachnospiraceae*, *Ruminococcaceae*, *Akkemansia*, *Prevotella*, *Ruminococcus*, *RF39*, *Sutterella*	Nagpal et al. (2018)

Although viruses are the most ubiquitous living species on the planet, their involvement is often overlooked as a component of the gut microbiome, which, as part of the “gut virome” (Liang and Bushman, 2021), is dominated by the bacteriophages *Caudovirales* and *Microviridae*. Individuals with elevated levels of *Caudovirales* and *Siphoviridae* in the gut perform better in executive functioning and verbal memory. In contrast, increased levels of *Microviridae* correlate with deteriorating executive functioning. Transplantation of microbiota from human donors with a high content of specific *Caudovirales* (>90% from the *Siphoviridae* family) improves the recognition of new objects in mice and upregulates genes affecting memory development in the prefrontal cortex suggesting that the gut virome is moving into focus as an important player in the gut-brain axis (Mayneris-Perxachs et al., 2022).

## 3 Interactions of gut microbiota with epithelia and gut function

The gut epithelium serves important functions, such as nutrient absorption, water and salt homeostasis, surveillance of luminal content as well as protection of the body by building a physicochemical barrier and collaborating with the immune system (Kim and Ho, 2010; Sommer and Backhed, 2013; Konig et al., 2016; Allaire et al., 2018a; Soderholm and Pedicord, 2019). The epithelium of the gut is made up of a monolayer of intestinal epithelial cells (IEC), absorptive enterocytes (EC, small intestine) or colonocytes (CC, colon), as well as enteroendocrine (EEC) and enterochromaffin cells (ECC), goblet cells (GC), paneth cells (PC), microfold cells (M cells; MC) or tuft cells (TC). IEC respond to luminal content, contribute to nutrient, water, and ion absorption, or sense and transfer information about luminal content including microbiota to the lamina propria for further processing, e.g., by cells of the innate and adaptive immune system or the nervous system (Nagler-Anderson, 2001; Rhee et al., 2009; Peterson and Artis, 2014; Konig et al., 2016; Allaire et al., 2018a; Kayama et al., 2020; Gershon and Margolis, 2021; Iftekhar and Sigal, 2021). An excerpt of the functions of IEC is listed in Table 2.

**Table 2:** Important functions of intestinal epithelial cells.

IEC	Function	Reviewed in:
Enterocytes and/or colonocytes	Absorption (nutrients, ions, and water), sensing of microbes, secretion of antimicrobials, barrier formation.	Wells et al. (2011), Yu et al. (2012), Kunisawa and Kiyono (2013), Allaire et al. (2018a), Lomax et al. (2019), Pelaseyed and Hansson (2020), Iftekhar and Sigal (2021), Seo et al. (2021), and Shu et al. (2023)
Enteroendocrine cells (EEC and ECC)	Hormone secretion; luminal sensing (e.g., nutrients, microbes, microbial metabolites); link in gut-brain axis; homeostasis (e.g., intestinal, metabolic, immune).	Peterson and Artis (2014), Arora et al. (2021), Hosseinkhani et al. (2021), Koopman et al. (2021), Xu et al. (2021), Osinski et al. (2022), Wei et al. (2022), Yu and Li (2022), Bayrer et al. (2023), and Meyer and Duca (2023)
Goblet cells (GC)	Secretion of mucins, antimicrobials, chemokines, and cytokines; antigen delivery to APC, barrier formation.	Pelaseyed et al. (2014), McCauley and Guasch (2015), Knoop and Newberry (2018), Allaire et al. (2018b), Hansson (2020), Fekete and Buret (2023), and Liu et al. (2023)
Microfold cells (MC)	Immunosurveillance; antigen sampling, transcytosis and transfer to APC; mucosal immunity.	Mabbott et al. (2013), Ohno (2016), Kimura (2018), Kobayashi et al. (2019), and Kanaya et al. (2020)
Paneth cells (PC)	Secretion of antimicrobials; sensing of microbes; efferocytosis; support of intestinal stem cells; mucosal immunity.	Lueschow and McElroy (2020), Barreto et al. (2022), Cui et al. (2023), and Wallaeys et al. (2023)
Tuft cells (TC)	Immunosurveillance; mucosal immunity; epithelial repair; chemosensory sentinel.	Schneider et al. (2019), Strine and Wilen (2022), and Bas et al. (2023)

APC, antigen presenting cells.

The lamina propria of the gut is innervated by primary afferent neurons (PANs), which are either of extrinsic (dorsal root ganglia and vagal) or intrinsic (enteric) origin (Gershon and Margolis, 2021). PANs can be activated by mechanical and chemical signals, and thus transfer information on the status and content of the gut to the nervous (enteric and central), immune, and hormone system, allowing the integration of signals to promote gut and whole-body homeostasis (Rhee et al., 2009; Abdullah et al., 2020; Gershon and Margolis, 2021; Sharkey and Mawe, 2023). Gut innervating nociceptor neurons for example support the mucosal barrier by influencing intestinal microbiota composition (Zhang et al., 2022) and by activating mucus secretion of goblet cells (Yang et al., 2022).

Microbial density and diversity increases along the longitudinal as well as the transverse axis of the gut, e.g., from the duodenum to the colon and the apical side of the epithelium to the lumen (Sekirov et al., 2010; Sommer and Backhed, 2013; Donaldson et al., 2016; Tropini et al., 2017). In the mouse colon for example, the outer but not the inner of the two mucus layers that cover the luminal side of the epithelium is colonized by bacteria (Johansson et al., 2008; Hansson, 2020). The gut epithelial barrier combines physical (e.g., tight intestinal epithelium, glycocalyx of intestinal epithelial cells, secreted mucus layers), chemical (for example secreted antimicrobials like C-type lectins, cathelicidins, defensins, lactoferrin, lysozyme, Lypd8, or secretory immunoglobulin A), immune and microbial barriers. Collectively, these barriers separate and protect the host from microbiota including pathogens, facilitate the entrapment and subsequent removal of pathogens by intestinal motility (peristalsis), contribute to the regulation of the microbiota population, and help maintain mucosal and immune cell homeostasis (Mavris and Sansonetti, 2004; Peterson and Artis, 2014; Hansson, 2020; Kayama et al., 2020; Shu et al., 2023). However, when the intestinal barrier is compromised, pathogens may breach the barrier leading to infectious and inflammatory diseases, as observed for example in inflammatory bowel disease (IBD) or pancreatitis (Shu et al., 2023). Pathogens employ various approaches to overcome the intestinal barrier, for example through pili and fimbriae to attach to epithelial cells, virulence factors like lipopolysaccharides, toxins, and enzymes, or the use of a secretion system to modify epithelial cells (Mavris and Sansonetti, 2004; Shu et al., 2023). Manipulation of the intestinal barrier by pathogens affects epithelial permeability for example via alterations of epithelial cell tight junctions, epithelial repair, or IEC renewal, differentiation, and apoptosis (Mavris and Sansonetti, 2004; Shu et al., 2023). The pathological condition of a compromised integrity of the intestinal barrier is called “leaky gut syndrome,” and is provoked by for example physical, environmental, or psychological stressors. The “leaky gut syndrome” is accompanied by a systemic pro-inflammatory response, bacterial translocation, and disturbed immune homeostasis (Kinashi and Hase, 2021; Álvarez-Herms et al., 2023). In more severe instances, this could potentially result in IBD or other clinically significant consequences (Camilleri, 2019).

Microbial sensing by gut epithelia is achieved by pattern recognition receptors (PRRs) expressed by epithelial and innate immune cells. PRRs such as toll-like (TLRs), NOD-like (NLRs), RIG-1-like (RLRs), or C-type lectin receptors (CLRs) recognize pathogen-associated or (more generally) microbe-associated molecular patterns (PAMPs, MAMPs) like flaggelin, peptidoglycans, lipopeptides, lipopolysaccharides (LPS), and others (Eckmann, 2004; Mavris and Sansonetti, 2004; Wells et al., 2011; Bishu, 2016; Coleman and Haller, 2017; Kayama et al., 2020; Coquant et al., 2021). Furthermore, bacterial quorum sensing molecules (QSM) that are secreted and utilized by bacteria to signal and collect information about the properties of their environment, as well as to influence gene expression and group behavior in a bacterial population, can directly or indirectly impact gut physiology (Coquant et al., 2021; Uhlig and Hyland, 2022; Oliveira et al., 2023). By affecting epithelial permeability, IEC viability, migration, and mucus production, as well as innate and adaptive immune cells QSM can for example influence barrier function and immune responses (Coquant et al., 2021; Uhlig and Hyland, 2022).

In addition to pattern recognition receptors described above, microbial sensing by the gut may also involve transient receptor potential (TRP) channels, taste receptors, and aryl hydrocarbon receptors (Najjar et al., 2020; Uhlig and Hyland, 2022). Bacterial signals sensed by IEC are relayed to the lamina propria via the release by epithelial cells of for example nucleotides, neurotransmitters, proteases, chemokines, and cytokines. Epithelial cell mediators may then directly or indirectly affect the innate and adaptive immune system as well as signaling by primary afferent neurons (Wells et al., 2011; Coleman and Haller, 2017; Lomax et al., 2019; Najjar et al., 2020).

## 4 Bidirectional interaction between gut and brain

Bidirectional brain-gut communication has a significant role in the regulation and modulation of functions of the GI tract, such as secretion, motility and permeability of the intestinal barrier, blood flow intensity, the immune activity of mucus membranes, as well as visceral sensations, in addition to pain. Additionally, the importance of enteric microbiota for brain functions is recently emerging (Rhee et al., 2009; Carabotti et al., 2015; Raskov et al., 2016; Martin et al., 2018; Cryan et al., 2019).

The bidirectional crosstalk involves microbiota colonizing the host surface (gastro-intestinal mucus membranes), organs such as exo- and endocrine glands, immune cells, afferent and efferent neurons, as well as different areas of the brain (Cryan and Dinan, 2012; Montiel-Castro et al., 2013). The brain controls gut function and the intraluminal milieu via the overall control over the autonomic and enteric nervous system modulating motility, secretion, and permeability in the GI tract, and this involves communication to cells in the lamina propria, smooth muscle cells, ECCs, neurons and immune cells in the intestinal wall (Collins et al., 2012; Kim and Shin, 2018; Jain et al., 2023).

## 5 Routes of communication between gut and brain

**Figure 1:**
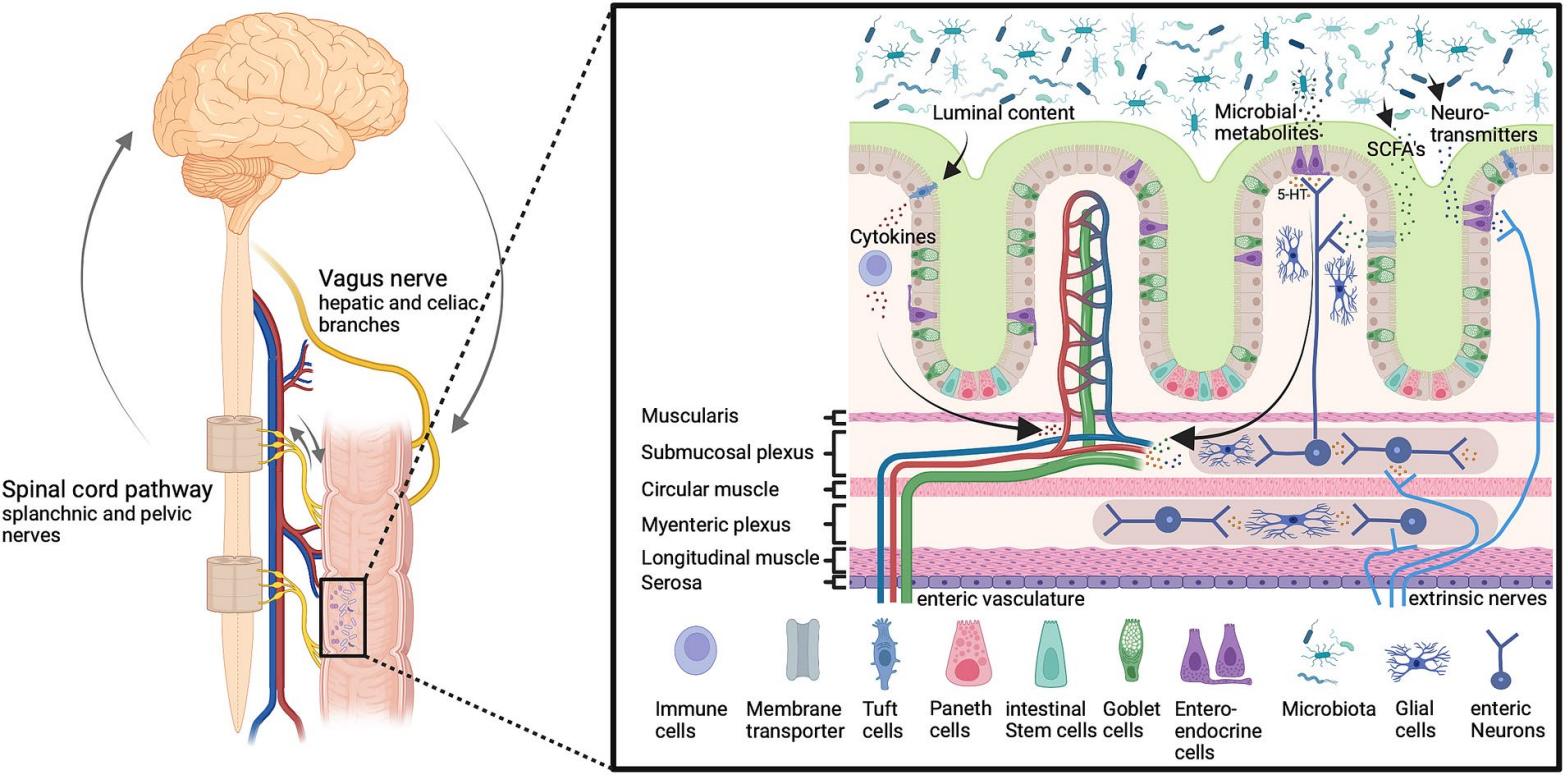
The microbiota-gut-brain-axis. Gut microbiota affect gut permeability and brain function through multiple humoral and neuronal signals and routes, including vagal and spinal afferents as well as circulating metabolites and immune cells. These pathways are important to maintain host homeostasis and gut function. Imbalances can lead to altered neuron function and information processing in the enteric, peripheral, and central nervous system and inflammation, which affects the host health status. Bioactive signals emerge from gut microbiota which releases a multitude of bioactive substances including neurotransmitters such as serotonin (5-HT), and short-chain fatty acids (SCFAs). Created with BioRender.com.

Bidirectional loops between the brain and the gut may involve neural, hormonal and immunological routes or a combination thereof (Bravo et al., 2011; Diaz Heijtz et al., 2011; Cryan and Dinan, 2012; Clarke et al., 2013; O’Mahony et al., 2014; Desbonnet et al., 2015; Chen et al., 2017; Jameson and Hsiao, 2018; Bassett et al., 2019; Cryan et al., 2019; Rincel et al., 2019; Mörkl et al., 2020; Rajendiran et al., 2021; Wen et al., 2021; Wang Y. et al., 2021; Connell et al., 2022; Figure 1).

### 5.1 Vagal afferent and efferent neurons

Visceral afferents innervating the GI tract either travel with the vagus nerve to the brain stem or with peripheral nerves to the spinal cord (Cryan et al., 2019). The vagus nerve contains 80% afferent and 20% efferent nerve fibers that bidirectionally connect the nucleus tractus solitarii (NTS), and the GI tract in a bottom-up and top-down fashion (Agostoni et al., 1957; Harrington et al., 2018; Cryan et al., 2019; Prescott and Liberles, 2022). Glutamatergic vagal afferents project into the NTS working as coordinators and sending feedback from the gut to the brain to the periphery (Altschuler et al., 1993; Cryan et al., 2019; Muller et al., 2020). Their sensory nerve terminals in the muscular layers and mucosa in the vicinity of EECs detect stretch, tension, as well as chemical signals (neurotransmitter, hormones, and metabolites), and transport sensory information from the intestine to the NTS (Berthoud et al., 2004; Furness et al., 2013; Appleton, 2018; Kaelberer et al., 2018; Ye et al., 2021). The efferent fibers connect the vagal dorsal motor nucleus with post-ganglionic and enteric neurons in the GI wall (Bonaz et al., 2021). Enteric neurons regulate peristalsis as well as secretion in the GI tract and the efferent vagal nerve fibers regulate immune cells and the release of inflammatory cytokines in the gut by the release of the neurotransmitter acetylcholine (Pavlov and Tracey, 2005; Chalazonitis and Rao, 2018). The immune cells interact with visceral sensory neurons and resident macrophages are the key immune cells responsible for β-endorphin secretion (Hughes et al., 2014; Veening and Barendregt, 2015). The bidirectional signaling via vagal connections is not only important for gut function but also plays an important role in mood regulation and host behavior (Klarer et al., 2014; Breit et al., 2018; Cryan et al., 2019; Bonaz et al., 2021). Severing the vagus nerve can have a detrimental impact on neurogenesis, impairing proliferation and causing a decline in the quantity of immature neurons in the hippocampus (O’Leary et al., 2018; Kelly et al., 2021).

### 5.2 Spinal visceral pathways

Due to a rostrocaudal shift in innervation ratio, the predominant afferent innervation of the proximal GI tract is provided through the vagus nerve, while the distal GI tract is mostly innervated by afferent spinal projections (Harrington et al., 2018). The lumbar splanchnic nerve innervates the proximal colon; the distal colon and rectum receive dual innervation both from the lumbar splanchnic and the sacral pelvic nerve, with cell bodies located in the thoracolumbar (TL, T10-L1) and lumbosacral dorsal root ganglia (DRG) (LS, L6-S2) (Ozaki and Gebhart, 2001; Brierley et al., 2018; Harrington et al., 2018). RNA sequencing revealed seven neuronal subtypes, with five of these subtypes almost exclusively abundant in TL, and the other two subtypes in LS spinal levels (Hockley et al., 2019).

DRG neurons projecting to the gut terminate there as intraganglionic laminar endings (IGLEs), intramuscular arrays (IMAs), or mucosal endings (Ozaki and Gebhart, 2001; Harrington et al., 2018; Prescott and Liberles, 2022). Mucosal terminals are important for defecation and stool passage control (Brierley et al., 2018). Approximately 80–100% of the proximal mesenteric but only 10–15% of distal colonic ganglia are innervated by IGLEs, which function as tension receptors. IMAs are located within the circular and longitudinal muscle layer and function as both tension and length receptors (Berthoud et al., 2004; Harrington et al., 2018). Serosa and mesentery are innervated by spinal afferents, the colorectal mesenteric by splanchnic, and the serosa by pelvic and splanchnic nerve fibers (Brierley et al., 2004; Beyak et al., 2006). Sensory nerve terminals are predominantly located in the mucosa, muscle, and serosa extending through the lamina propria into crypts and villi (Berthoud et al., 1995; Beyak et al., 2006; Powley et al., 2011). Peptidergic neurons expressing calcitonin gene-related peptide (CGRP+) are predominantly found in the circular muscle, myenteric ganglia, and submucosa, whereas nonpeptidergic nerve endings (CGRP-) mainly innervate the mucosal crypts, myenteric ganglia, and submucosa suggesting different functions (Brookes et al., 2013; Spencer et al., 2022). Multiple serotonin (5-HT) receptor subtypes are located on spinal afferent nerve terminals in the different layers of the intestine and contribute to the communication with ECCs (Dodds et al., 2022), including 5-HT_1-4_ and 5-HT_7_, which are distributed across intrinsic and extrinsic afferent nerve fibers, smooth muscle cells, enterocytes, and immune cells. Among the most studied receptors, the 5-HT_3_ and 5-HT_4_ receptors play a pivotal role in gut motility, pain sensation, transit time, and sensitivity. 5-HT_3_ receptors are mainly expressed by nerve fibers in the submucosa extending into the mucosal layer in close proximity to the ECCs acting as sensors for signals emerging from the ECCs (Mawe and Hoffman, 2013; Kim and Khan, 2014; O’Mahony et al., 2015). ECCs secrete approximately 90% of the body’s 5-HT and via neuronal loops affect gut motility, secretion, and sensitivity by sensing not only nutrients and commensal bacteria but also pathogens, infections, and inflammatory processes (Bellono et al., 2017).

Spinal afferent neurons also serve efferent functions affecting vascular permeability, blood flow, motor reactions, or mucus secretion by releasing neuropeptides such as CGRP or Substance P upon activation (Holzer, 2006). The extrinsic efferent neural pathways regulating the intestine involve the autonomic nervous system, especially sympathetic spinal adrenergic efferents (Andrews and Ashley Blackshaw, 2010). The axons of the efferent fibers run in parallel to those of sensory afferents, with their cell bodies located in the lateral horn of the thoracolumbar spinal cord and traveling through the ventral roots to the abdominal prevertebral sympathetic ganglia (celiac, superior mesenteric, and inferior mesenteric ganglia), projecting to muscles, glands, and target organs (Wood, 2004; Gaman and Kuo, 2008; Kyloh et al., 2022). Due to the highly complex innervation, it has been difficult to experimentally dissect the precise contributions of spinal and vagal pathways within a living animal (Berthoud and Neuhuber, 2000; Sengupta, 2009; Kyloh et al., 2022). Only recently and with the development of novel tracing techniques and optogenetics, thoracolumbar and lumbosacral afferents to the gut are emerging as important for gut innervation, function, and visceral pain (Hibberd et al., 2018; Spencer and Hu, 2020; Kyloh et al., 2022). Nevertheless, comprehensive research is still needed to elucidate the complex interaction routes. Current investigations focus on the neuroimmune axis as well as on the release of neuropeptides among other immune mediators (Holzer and Farzi, 2014; Udit et al., 2022). Direct signaling of neurons can activate immune cells and thereby modulate inflammatory reactions (Udit et al., 2022). Transient receptor potential vanilloid 1 (TRPV1) expressing nociceptor neurons act on the enteric nervous system, as well as intestinal epithelial cells, thereby modulating gut permeability, microbiota abundance, and eliciting the release of signaling molecules by microbiota (Holzer and Farzi, 2014; Udit et al., 2022).

### 5.3 Humoral pathways

Chemical mediators such as chemokines, neuropeptides, neurotransmitters, endocrine messengers, cytokines, exotoxins, short-chain fatty acids (SCFAs), and other metabolites travel with the blood or lymphatic system (Cryan et al., 2019; Uhlig et al., 2020). The composition of microbiota is directly linked to the generation of SCFAs and each bacterial class generates specific SCFAs. SCFA metabolites not only exert local effects but also affect the host’s glucose homeostasis, satiety, immune system, and brain signaling (Crawford et al., 2022). Dysbiosis can be caused by an increase in sympathetic activity in the gut as a result of acute and chronic stress, which disrupts the intestinal barrier by activating mast cells through corticotropin-releasing hormone, which in turn allows antibodies, microbial metabolites, toxins, and lipopolysaccharides in the gut to enter the systemic circulation (Wallon et al., 2008; Kim and Shin, 2018; Toral et al., 2019; Shu et al., 2023). An important barrier for the humoral communication between the gut and the brain is the blood–brain barrier (BBB), and the significance of gut microbiota for BBB development has recently been shown in germ-free mice, who develop an increased BBB permeability which is restored by the recolonization of microbiota (Cryan et al., 2019). The hypothalamic–pituitary–adrenal (HPA) axis is related to psychological and physical stress and regulates multiple physiological systems including the gut permeability, causing bacterial translocation, and release of messenger substances, which affects the intestinal barrier and BBB permeability (Vagnerová et al., 2019; Geng et al., 2020). GF mice show decreased tyrosine (the rate-limiting substrate of noradrenaline and dopamine synthesis) and increased catecholamine levels which imply that gut microbiota modulate dopamine and noradrenaline turnover in the brain (Matsumoto et al., 2013; Nishino et al., 2013). Interestingly, SCFAs can pass the BBB under healthy conditions whereas neurotransmitters such as gamma-aminobutyric acid (GABA) and 5-HT do not pass in a healthy state, however, this may change during inflammation, allowing them to enter the CNS (Takanaga et al., 2001; Margolis et al., 2021; Modesto Lowe et al., 2023).

## 6 Metabolites and neurotransmitters secreted by gut microbiota

One of the main functions of intestinal bacteria is to help in food digestion and production of micronutrients that the human organism cannot synthesize on its own (Park and Im, 2020). Host species-specific characteristics of the gut microbiota in some of the most common animal models may reflect differences in host factors, such as diet, genetic background, sex, and age (Nagpal et al., 2018). For example, fecal levels of lactate are higher in mice, while acetate and propionate levels are highest in human feces (Nagpal et al., 2018). These differences may contribute to the translational gap between mice and men specifically regarding modeling brain functions and neuropsychiatric disorders.

### 6.1 Neurotransmitters

Bacteria produce a multitude of neurotransmitters and biologically active substances. Secondary bile acids and other metabolites are formed from primary bile acids produced by the liver in the intestine as a result of metabolism by the intestinal microbiota (Jiao et al., 2018; Funabashi et al., 2020). Bile acids can control neurotransmitter receptor functions, e.g., muscarinic acetylcholine and GABA receptors, and protect from neurodegeneration (Kiriyama and Nochi, 2019). Several strains of gut bacteria synthesize and release substances acting as or like neurotransmitters such as GABA, 5-HT, tryptamine, acetylcholine, L-dopa, norepinephrine, or histamine (Table 3; Kim et al., 2018). Of these, glutamate acts as a main excitatory neurotransmitter in the central nervous system (Henter et al., 2021), and excitatory amino acids associated with *Lactobacillus* are discussed as key factors in anxiety-like behavior (Oroojzadeh et al., 2022). The interactions along the gut-brain axis are bidirectional (Zhang et al., 2023) and for example, endogenous cannabinoids not only target neurons and immune cells but also play a major role in metabolism, energy homeostasis, and as regulators of the crosstalk between gut microbiota and host metabolism (Oroojzadeh et al., 2022; Zhang et al., 2023). However, the mutual crosstalk and feedback mechanisms between the CNS and the gut microbiota are still incompletely understood.

**Table 3:** Metabolites/neurotransmitters produced by the gut microbiome.

Metabolite/neurotransmitter	Microbiota species	References
Acetylcholine	*Lactobacillus* spp.	Kawashima et al. (2007)
Cannabinoid anandamide (AEA)	*Lactobacillus johnsonii* and *Lactobacillus reuteri*	Petrie et al. (2021)
Dopamine (DA)	*Lactobacillus* spp.	Kawashima et al. (2007), Ozogul (2011), Kuley et al. (2012), and Holzer and Farzi (2014)
Gamma aminobutyric acid (GABA)	*Lactobacillus* spp.	Takanaga et al. (2001), Bravo et al. (2011), Barrett et al. (2012), Janik et al. (2016), and Kim and Shin (2018)
	*Bifidobacterium genus*, *Lactobacillus* spp.	Socała et al. (2021)
	*Lactobacillus brevis*, *Bifidobacterium dentium*	Barrett et al. (2012)
Histamine	*Lactobacillus* spp.	Landete et al. (2008) and Hemarajata et al. (2013)
↓ Proinflammatory cytokine TNF-α	*Lactobacillus* spp.	Thomas et al. (2012)
Serotonin (5-HT)	*Streptococcus* spp., *Enterococcus* spp., *Escherichia* spp.	Dinan et al. (2013)
	*Escherichia* spp., *Hafnia* spp., *Klebsiella* spp., *Lactobacillus* spp., *Morganella* spp., *Streptococcus* spp.	O’Mahony et al. (2015)
Short-chain fatty acids (SCFAs): acetic acid, butyric acid, propionic acid	*Lactobacillus* spp., *Bacteroides* spp., *Clostridiae* spp.	Turnbaugh et al. (2009), Collins et al. (2015), Bull-Larsen and Mohajeri (2019), and Park and Im (2020)
	*Lactobacillus brevis* and *Bifidobacterium dentium*	Barrett et al. (2012)
Tryptophan	↑ Concentrations in the blood of GF male animals suggest a humoral mechanism of the microbiota influencing CNS serotonergic neurotransmission.	Cryan and Dinan (2012)

### 6.2 Non-coding RNAs

Only recently, non-coding RNA species, and in particular microRNA (miRNA), are emerging as hub regulators of entire gene sets that are essential for many cellular functions including pluripotency and developmental processes (Lagos-Quintana et al., 2001; Landgraf et al., 2007; Zeidler et al., 2021). In fecal samples from mice and humans, miRNAs are detectable in large quantities, with HoxP-positive cells being the main source (Liu et al., 2016). Gene regulation mediated through fecal miRNA enables the host to exert control over the gut microbiota (Liu et al., 2016). Several miRNAs (miR-515-5p and miR-1226-5p) are found in bacteria (*Escherichia coli* and *Fusobacterium nucleatum*), and they selectively affect bacterial gene transcripts and regulate bacterial growth (Liu et al., 2016). In mice with IEC-specific conditional depletion of miRNAs, there is an imbalance of the gut microbiota with symptoms of colitis, and transplantation of healthy fecal miRNAs restores fecal microbes and improves the course of colitis, indicating a major role of miRNAs for the bidirectional communication between host and microbiota in the gut (Liu et al., 2016).

## 7 Disturbed gut-brain communication and disease

The composition of the mammalian gut microbiome is critically important for the development of neural circuits that are involved in emotional processing, motor control, learning, and memory. Enteric microbiota can communicate with the host through several mechanisms, affecting epithelial cells, ECCs, and neurons (Carabotti et al., 2015; Cryan et al., 2019) and contribute to shape the intestinal permeability, motility, and mucus production (Yan and Kentner, 2017; Ait-Belgnaoui et al., 2018; Li R. et al., 2022). An altered quantitative and qualitative composition of the gut microbiome can induce the production of metabolites with cytotoxic effects, promote neuroinflammation, and disrupt immune cell function. This results in the inhibition of synaptic transmission and gamma oscillations in the hippocampus, a brain region that plays a crucial role in innate and cognitive behavior (Çalışkan et al., 2022; Connell et al., 2022). Studies in GF mice document the importance of bacterial colonization of the gut after birth for the development of brain functions and expression of miRNAs and messenger RNAs in the hippocampus that cannot be reversed by colonization of gut microbiota in adolescent mice (Chen et al., 2017). In the CNS, neurotransmission is profoundly disturbed in the absence of a normal gut microbiome (Clarke et al., 2013). GF mice exhibit increased exploratory and risk-taking behaviors as well as hyperlocomotion, and these behaviors are determined by early but not late bacterial colonization (Diaz Heijtz et al., 2011; Neufeld et al., 2011). As a consequence, the brain’s chemistry differs from that of “normal” mice with region-specific changes in the expression of 5-HT and brain derived neurotrophic factor (BDNF) (Wrase et al., 2006; Sampson and Mazmanian, 2015; Yano et al., 2015; Bauer et al., 2016; Sharon et al., 2016). Additionally, heightened levels of proteins that regulate the maturation and functionality of neural synapses, such as the synaptic vesicle glycoprotein synaptophysin and postsynaptic density protein 95 (PSD95), are present in the striatum of GF mice which show alterations in spatial working memory and reference memory, indicating impairment of the development of the hippocampus (Diaz Heijtz et al., 2011; Gareau et al., 2011; Glinert et al., 2022). The regional specificity suggests that the pathways underlying described diversities are specifically important for various brain regions, or that the timing of bacterial influences may vary across different areas of the brain (Collins et al., 2012). Microbiota seem to be crucial for the formation of stress response related brain circuits, while emotional and physiological stress affects the gut (Kim and Shin, 2018). Stress causes dysbiosis, which consecutively leads to altered synthesis of biologically active substances, including neurotransmitters (Bassett et al., 2019).

For instance, only 2 h of social separation alters the quantitative and qualitative composition of the gut microbiota in mice and leads to a decline in the *Lactobacillus* population (Galley et al., 2014). A brief disruption of the intestinal microbiota composition by administration of the antibiotic vancomycin has a significant effect on physiological or behavioral parameters in later life (Cryan and Dinan, 2012). Mechanistically, all mental disorders in Table 4 are associated with a leaky gut, neuroinflammation, and hyper-activated microglial cells, for which gut-residing bacteria and their metabolites are important contributors. Respectively, patients show a shift towards pro-inflammatory colonic microbiota, harboring more Gram-negative bacteria containing lipopolysaccharides (LPS) which can cause inflammatory reactions. It is also known that bacteria with pro-inflammatory properties, such as *Alistipes*, *Eggerthella*, and *Flavonifractor*, are found in greater numbers, whereas the number of bacteria with anti-inflammatory properties, in particular *Bifidobacterium* spp., *Coprococcus*, *Eucbacterium*, *Eubacterium rectale*, *Faecalibacterium*, *Faecalibacterium prasunitzii*, *Lactobacillus* spp., *Prevotella*, *Roseburia*, is decreased compared to healthy people. Various metabolites, mainly SCFAs, as well as bacterial metabolites, including neurotransmitters (acetylcholine, dopamine, norepinephrine, GABA, glutamate, 5-HT), are involved in the pathogenesis (Eicher and Mohajeri, 2022). Increasing evidence suggests that the gut microbiota may contribute to the pathogenesis of Alzheimer’s disease (AD) as a source of amyloid proteins.

**Table 4:** Association of mental/neurological disorders with microbiota, metabolites, or neurotransmitter changes.

Disorder	Associated microbiota	Metabolite/neurotransmitter change/mechanism	Reference
Attention-deficit-hyperactive disorder (ADHD)	*Lactobacillus* spp., and *Bifidobacterium* spp.	Tryptophan ↑ SCFAs ↑ Polyunsaturated fatty acids ↓ Dopamine	Barrett et al. (2013), Dinan et al. (2013), Erny et al. (2015), O’Mahony et al. (2015), Pärtty et al. (2015), and Bassett et al. (2019)
	*↑ Bifidobacterium genus*	It was assumed that the increase of Bifidobacterium was linked to significantly enhanced 16S-based predicted bacterial gene functionality encoding cyclohexadienyl dehydratase, the enzyme that is involved in the synthesis of phenylalanine (precursor of DA).	Aarts et al. (2017)
	*Enterococcus* spp., *Escherichia* spp., and *Streptococcus* spp.	↓ 5-HT	Bull-Larsen and Mohajeri (2019)
	*Bifidobacterium* spp., *Enterococcus* spp., *Escherichia* spp., *Lactobacillus* spp., *Clostridia* spp., *Streptococcus* spp.	↓ 5-HT	Dam et al. (2019), Boonchooduang et al. (2020), and Eicher and Mohajeri (2022)
	*↑ Actinobacteria (genus Bifidobacterium)* *↓ Firmicutes*	Compensatory ↑ DA	Aarts et al. (2017)
Autism spectrum disorder (ASD)	*↓* fermenting bacteria: *Coprococcus*, *Prevotella*, and *Veillonellaceae*		Kang et al. (2013)
	*↑ Bacteroidetes*, *Proteobacterium*, *Desulfovibrio* species and *Bacteroides vulgatus*; *↓ Bifidobacterium genus*, *Firmicutes* and *Actinobacterium*	LPS-induced inflammation LPS decreases levels of glutathione, an important antioxidant involved in heavy metal detoxification in the brain	Zhu et al. (2007) and Finegold et al. (2010)
	*Hespellia*, *Anaerostipes*, *Desulfovibrio* spp.		Finegold et al. (2010)
Alzheimer’s disease (AD)	*Escherichia coli*, *Bacillus subtilis*, *Mycobacterium tuberculosis*, *Salmonella enterica*, *Salmonella typhimurium*, *Staphylococcus aureus*	↑ Bacterial amyloids production	Jiang et al. (2017), Megur et al. (2021), Tran and Mohajeri (2021), and Eicher and Mohajeri (2022)
Anxiety-like behavior	*Lactobacillus* spp.	Glutamate is a key excitatory neurotransmitter in the CNS and excitatory amino acids	Henter et al. (2021)
	*Bifidobacterium dentium*, *↓ Lactobacillus brevis*	↓ GABA	Barrett et al. (2012)
Bipolar disorder (BD)	*Toxoplasma gondii*	Chronic inflammation	Sutterland et al. (2015)
	↑ *Bifidobacterium*, *Oscillibacter*, *Enterococcus*, *Flavonifractor, Streptococcus* and *Megasphaera*; ↓ *Roseburia*, *Faecalibacterium*, and *Ruminococcus*		McGuinness et al. (2022)
Fibromyalgia	↓ Diversity of bacteria; ↓ *Bifidobacterium* and *Eubacterium genera*	Altered levels of glutamate and serine	Clos-Garcia et al. (2019)
	↓ *Bacteroides thetaiotaomicron*, *Bacteroides uniformis*, *Prevotella copri*; ↑ *Clostridium scindens*, *Enterocloster bolteae*	↓ α-Muricholic acid and other secondary bile acids	Minerbi et al. (2023)
Major depressive disorder (MDD)	*↓ Coprococcus* spp. and *Dialister*	↓ SCFAs	Valles-Colomer et al. (2019), Socała et al. (2021), and Modesto Lowe et al. (2023)
	↑ *Flavonifractor*, *Escherichia*/*Shigella* and *Veillonella*; ↓ *Prevotella* and *Ruminococcus*	↑ Bacteria associated with glutamate and GABA metabolism and ↓ bacteria producing SCFA(e.g., butyrate)	McGuinness et al. (2022)
	↑ *Lactobacillus*, *Streptococcus*, and *Enterococcus*	↑ Increased lactic acid	Valles-Colomer et al. (2019) and McGuinness et al. (2022)
	↓ *Faecalibacterium* and *Coprococcus*	↓ SCFAs (mainly butyrate)	
Migraine	↓ Firmicutes family: *Clostridial* Clusters IV and XIVa, *Coprococus* spp., *Eubacterium hallii Faecalibacterium prausnitzii*, *Lachnosiraceae* spp., and *Roseburia* spp.	↓ 5-HT ↓ SCFAs (mainly butyrate)	Kappéter et al. (2023)
	*Akkermansia mucinophila*, *Alistipes putredinis*, ↓ *Bacteroides vulgatus* and *uniformis*, *Prevotella copri*, *Roseburia inulinivorans*, *Veilonella* spp.	↓ Propionate synthesis and BBB protection from oxidative stress	
	↑ *Alcaligenes* spp., *Candida* spp., *Clostridium coccoides* and *propionicum*, *Eggerthella lenta*, *Micromycetes* spp., *Pseudonocardia* spp., and *Rhodococcus* spp.		Kopchak and Hrytsenko (2022)
	↑ *Bacteroides* and *Coprococcus* ↓ *Prevotella* and *Escherichia*-*shigella*	↓ L-tryptophan, linoleic acid, and nicotinamide; ↑ L-arginine, glutamic acid, L-tyrosine, L-DOPA, 3-indoxyl sulfate	Wen et al. (2019)
Neuropathic pain	*↑ Lactobacillus*	41 Upregulated metabolites and 31 downregulated metabolites, among these, differentially expressed metabolites including allantoin, D-quinovose and D(−)-beta-hydroxy butyric acid, N6,N6,N6-trimethyl-l-lysine, 3-methylhistidine, exhibited consistent expression trends. The lower level of 2-hydroxybutyric acid was in both serum and spinal cord samples from CCI rats in comparison to sham rats	Chen et al., 2021
	*↑ Lactobacillus*	↑ SCFAs (propionate, and butyrate)	Zhou et al. (2022)
Parkinson’s disease (PD)	*↑ Bacteroidetes*, *Proteobacteria*, and *Verrucomicrobia*; *↓ Firmicutes*	↓ SCFAs chronic systemic inflammation	(Shannon, 2022)
	*↓ Genera Blautia*, *Coprococcus*, and *Roseburia* (butyrate-producing bacteria with anti-inflammatory properties) *↑ Proteobacteria* (genus *Ralstonia*) with proinflammatory properties	↓ SCFAs	(Keshavarzian et al., 2015)
Schizophrenia (SZ)	*Succinvibrio* and *Corynebacterium*	Association with the severity of symptoms	Li et al. (2020)
	↑ *Prevotella*, *Megasphaera*; ↑ *Escherichia*/*Shigella* and *Veillonella*; ↓ *Bacteroides*, *Haemophilus*, *Roseburia*, and *Streptococcus*		McGuinness et al. (2022)
	*Bacteroides*, *Prevotella*, and *Clostridium* are among the top 3 altered genera, *Bacteroides-Prevotella* ratio ↑	↑ SCFAs	Nguyen et al. (2019) and Li et al. (2023)
	*Ruminococcus*		Li et al. (2020)
	*Blautia*		Shen et al. (2018)
	*Toxoplasma gondii* can cause a risk of mania developing	Chronic inflammation	Dickerson et al. (2014)
Stroke	↑ *Enterobacteriaceae* and *Prevotella*; *↓* SCFA-producing bacteria; *↓ Lachnospiraceae* and *Ruminococcaceae*; *↓ Firmicutes* and *Faecalibacterium*; *↓↑ Bacteroidetes*	↑ LPS, ↓ Butyric acid, ↓ SCFAs	Benakis and Liesz (2022)
	↑ *Enterobacteriaceae* ↓ *Clostridium tyrobutyricum*	↑ LPS, ↓ Metabolites of the tryptophan-kynurenine pathway and ↑ indole metabolites, impairing the integrity of BBB; ↓ SCFAs and bile acids	Zeng et al. (2023)
Traumatic brain injury		↑ Metabolites concerned with late glycolysis, cysteine, and one carbon metabolites, as well as metabolites affected by arginine metabolism, endothelial dysfunction, and responses to hypoxia	Coleman et al. (2023)
	7-Day post-TBI: ↑ *Streptococcus* (*Streptococcaceae*) ↓ *Akkermansia (Verrucomicrobia)*	↓ Bacterial secretion system, sulfur metabolism, biosynthesis of steroids, no-homologous end-joining, and protein processing in the endoplasmic reticulum; ↑ Epithelial cell signaling in *Helicobacter pylori* infection and pentose as well as glucuronate interconversions; ↑ Indole-3-acetaldehyde (IAAld) and indole-3-ethanol (IEt); ↑ 5-HT; ↓ Indole-3-lactic acid (ILA) and skatole; ↓ Melatonin and 5-hydroxy indole acetic acid (5-HIAA); Tryptophan metabolism through the ↑ kynurenine (KYN) and ↓ neuroprotective kynurenic acid (KYNA); ↓ Xanthurenic acid (XA); ↑ KYN/Tryptophan and ↓ KYNA/KYN correlation indicates increased metabolism through the neurotoxic pathway	Zheng et al. (2022)
	28-Day post-TBI: ↑ *Streptococcus* (*Streptococcaceae*), *Proteobacteria*, *TM7* and *Actinobacteria*; *↓Verrucomicrobia*, *Bacteroidetes*, *Cyanobacteria*, and *Deferribacteres*	↓ Gut microbiota functions of biosynthesis, including lipopolysaccharide, *n*-Glycan, primary and secondary bile acid, and steroids; ↑ Metabolism of chlorophyll, glycerophospholipid, thiamine, porphyrin, and riboflavin; ↑ 5-HT; ↑ Tryptophan metabolism through the kynurenine KYNA is often considered to be neuroprotective; ↑ The ratio KYNA/KYN; ↓ Melatonin, 5-HIAA and XA	Zheng et al. (2022)
Visceral pain	↑ Phylum *Bacteroidetes*, *Proteobacteria*, and *Tenericutes*; *↓* Phylum *Firmicutes* and *Actinobacteria*	In rats aged 4 and 8 weeks during 4 and 6 weeks after vancomycin administration in dose 100 mg/kg	(O’Mahony et al., 2014)
		Activation of the immune, humoral, and neuroendocrine (hypothalamic–pituitary–adrenal axis) systems, both autonomic (*nervus vagus*) and enteric nervous systems, spinal afferents nerves, 5-HT, SCFAs, tryptophan-related metabolites, and neurometabolites (dopamine, GABA, noradrenaline) potentially modulating function of CNS Histamine produced by microbiota and visceral pain	Moloney et al. (2015, 2016), Agirman et al. (2021), and De Palma et al. (2022)

Despite the growing body of evidence linking dysbiosis with mental disorders such as schizophrenia (SZ), our understanding of the functional consequences of the gut microbiota and their influence on metabolite quantities in the blood and tissue of patients remains limited (Li et al., 2020, 2023). Metabolites such as butyric acid which is detectable in human breath gas are emerging for diagnosing SZ and major depressive disorders (Henning et al., 2023).

### 7.1 Gut microbiome and depression

Depression is a heterogeneous mood disorder with a complex yet not sufficiently understood neurobiology that has strong links to a dysfunction of the microbiome-gut-brain axis (Gheorghe et al., 2022). Clinical studies have found differences in the composition of the gut microbiota in patients with depression compared to individuals without mental disorders (Valles-Colomer et al., 2019; Socała et al., 2021; Green et al., 2023; Modesto Lowe et al., 2023). Common to all studies is an increase in the number of lactic acid-producing bacteria such as *Lactobacillus*, *Streptococcus*, and *Enterococcus*, and a decrease in the number of bacteria producing SCFAs (mainly butyrate) such as *Faecalibacterium* and *Coprococcus* (Table 4) (Valles-Colomer et al., 2019; McGuinness et al., 2022). There is evidence of a correlation between certain gut bacteria and depression symptoms (Simpson et al., 2021; McGuinness et al., 2022). However, their involvement in the pathophysiology of the mental disorder is not well understood (Green et al., 2023). Meta-analyses suggest that probiotics as an adjunctive treatment may reduce depressive symptoms, (Chao et al., 2020; El Dib et al., 2021; Green et al., 2023). However, probiotic preparations to date are limited to a single bacterial strain or, at best, a small number of strains (Green et al., 2023). In contrast, fecal microbiota transplantation (FMT), which encompasses the complete human gut microbiome containing thousands of potentially symbiotic strains (Strandwitz et al., 2019), may be better suitable since it alters the composition of the gut microbiota more effectively (Green et al., 2023).

One of the links in the pathogenesis of major depressive disorder (MDD) in men may be low testosterone levels, which is associated with disturbed gut microbiota, and as a result, with impaired functioning of the gut-brain axis (Dwyer et al., 2020; Gheorghe et al., 2022). *Mycobacterium neoaurum* produces the enzyme 3-beta-hydroxysteroid dehydrogenase (3β-HSD) and this may represent a new link between gut dysbiosis and depression in particular in men (Gheorghe et al., 2022; Li D. et al., 2022).

Thus, the mechanisms of MDD development involve the interaction of many components of biological origin, including the microbiota and gut-brain axis. Therefore, therapeutic and prophylactic strategies aiming at correcting the intestinal microbiome, such as prebiotics/probiotics, and FMT, are considered promising treatment options for MDD (Donoso et al., 2023).

## 8 Gut microbiome and pain disorders

Disruption of microbiota colonization caused by antibiotics in early life is not only associated with mental disorders but also visceral hypersensitivity and altered spinal cord signaling in adults (O’Mahony et al., 2014). Temporary changes in the gut microbiome composition during the critical period in newborn rats have long-term effects on nociceptive pathways, and the maturating pain system is influenced by the microbiome (Cryan and Dinan, 2012). Although the mechanisms as well as their up- and down-stream signaling pathways are largely unknown, there is a growing focus on the role of intestinal microbiota and their metabolites in neuropathic pain disorders (Li R. et al., 2022). The microbiota content in the gut is altered in rodent injury models of neuropathic pain (Chen et al., 2021). Specifically, the abundance of *Lactobacillus* phyla is significantly increased in the gut and accompanied by changes in serum and spinal cord metabolites (Table 4). Recent studies support a link between intestinal microorganisms and neuropathic pain in patients (Guo et al., 2019; Ding et al., 2021; Li R. et al., 2022; Zhang et al., 2023) suggesting, that alterations of specific microbiota strains are causally involved in metabolic disturbances associated with neuropathic pain. Microbiota-derived LPS, SCFAs, peptidoglycans, trimethylamine, or secondary bile acids affect neurons and non-neuronal cells along the pain pathway like immune cells, microglia, or astrocytes, resulting in elevated plasma levels of pro-inflammatory cytokines (IL-1β, IL-6, and TNF-α), chemokines (CCL2 and CXCL1), anti-inflammatory IL-4 as well as neuropeptides and opioids (Cryan et al., 2019; Park and Kim, 2021; Liu et al., 2022). Metabolites or even short RNAs directly act on receptors and ion channels (GABA receptors, TLRs, TRP channels, acid-sensitive ion channels) expressed by nociceptive primary afferents, and induce nociceptor activation and sensitization (Ji et al., 2014, 2016; Lutz et al., 2014; Chow and Gulbransen, 2017; Chiu, 2018). Evoked intestinal permeability increases the levels of pro-inflammatory factors circulating in the blood plasma, and the intensity of pain (Piche et al., 2009; Ernberg et al., 2018). Several of the above-mentioned inflammatory factors penetrate through the BBB and may even act on circuits in the spinal dorsal horn or brain areas processing painful stimuli (Varatharaj and Galea, 2017; Zhu et al., 2018; Çalışkan et al., 2022). As a consequence bacteria can alter emotional, motivational, and cognitive functions giving rise to mental comorbidities of pain such as depression or sleep disturbance (Takanaga et al., 2001; Kawashima et al., 2007; Bravo et al., 2011; Ozogul, 2011; Barrett et al., 2012; Kuley et al., 2012; Holzer and Farzi, 2014; Janik et al., 2016; Kim and Shin, 2018; Li R. et al., 2022). On the other hand, antinociceptive effects of certain microbiota are emerging such as *Lactobacillus reuteri* targeting the nociceptive transducer ion channel TRPV1, and visceral antinociceptive effects are emerging for probiotic *B. infantis* 35,624 (Mckernan et al., 2010; Perez-Burgos et al., 2015).

A second pain disorder with strong links to the gut microbiome is migraine, and migraine pathophysiology involves the 5-HT pathway or SCFAs (Arzani et al., 2020; Lanza et al., 2021; Crawford et al., 2022; Kappéter et al., 2023). SCFAs reduce hyperalgesia and decrease the release of TNFα and IL1-β in the gut in a rodent migraine model (Crawford et al., 2022). Therefore, the loss of 5-HT and SCFAs producing bacteria in the gut, such as the Firmicutes family (*Faecalibacterium prausnitzii*, *Coprococus* spp., *Roseburia* spp., *Lachnosiraceae* spp., *Clostridial* Clusters IV and XIVa, and *Eubacterium hallii*) is considered a highly important factor in migraine pathogenesis (Kappéter et al., 2023). Another important link is the intestinal propionate synthesis and BBB protection from oxidative stress due to the decrease of *Akkermansia mucinophila*, *Alistipes putredinis*, *Bacteroides vulgatus* and *uniformis*, *Prevotella copri*, *Roseburia inulinivorans*, and *Veilonella* spp. (Table 4; Kappéter et al., 2023), with probiotic dietary supplements or FMT effectively decreaseing the frequency and intensity of migraine attacks (Crawford et al., 2022; Kappéter et al., 2023). These discoveries paved the way for the development of personalized migraine therapies based on the microbiome (Crawford et al., 2022).

Microbiota dysbiosis also contributes to the pathogenesis of visceral pain in irritable bowel disease which affects between 5 and 10% of the general population worldwide and involves multiple processes including immune, humoral and neuroendocrine (HPA axis) factors, autonomic (nervus vagus) and enteric nervous systems, spinal afferents nerves, 5-HT, SCFAs, tryptophan-related metabolites, gut hormones, and neurometabolites (dopamine, GABA, noradrenaline) (Moloney et al., 2015, 2016; Agirman et al., 2021). After vancomycin administration *Bacteroidetes*, *Proteobacteria*, and *Tenericutes* increase, and the phylum *Firmicutes* and *Actinobacteria*, decreases in animals (Table 4). Temporary changes in the composition of the GI microbiota during a critical developmental period have long-term effects on nociceptive pathways, and the gut microbiome in particular in male newborns but not adult rats (O’Mahony et al., 2014). Histamine is emerging as a relevant mediator of visceral hyperalgesia (De Palma et al., 2022), and multiple studies investigating the highly complex processes are well summarised in several recent review articles (Alizadeh et al., 2022; Ustianowska et al., 2022; Mayer et al., 2023; Pujo et al., 2023; Sarnoff et al., 2023; Shaikh et al., 2023; Shin and Kashyap, 2023).

## 9 Sex-specific differences

In general, men are more prone than women to develop brain and nervous system disorders with impaired synthesis of neuroactive substances, and the male gender is a significant risk factor for delirium following surgery (Wang H. et al., 2021; Wittmann et al., 2022; Van Pee et al., 2023). This may be related to disturbed gut microbiota potentially due to unhealthy lifestyles which are more common in the male gender (Rincel et al., 2019; Wang H. et al., 2021; Gamage et al., 2023). Increasing evidence points to differences in the gut microbiota composition, neuronal processing in the CNS, and the HPA axis between men and women that may be related to respective differences in cognitive strategies and brain function in health and disease (Sisk-Hackworth et al., 2023). Male GF mice, in comparison to conventionally colonized control animals, exhibit a significantly higher hippocampal concentration of 5-HT and its main metabolite 5-hydroxyindoleacetic acid, in contrast to immunological and neuroendocrine manifestations that occur in both genders (Clarke et al., 2013; Rieder et al., 2017). The amount of tryptophan, a precursor of 5-HT, is increased in the blood of male GF rodents, indicating a humoral pathway through which microbiota can affect serotonergic neurotransmission in the CNS (Clarke et al., 2013). Interestingly, the post-weaning colonization of GF mice with gut microbiota could not rescue the neurochemical alterations caused by the absence of microorganisms in early life, although peripheral tryptophan availability was restored and changes in anxiety-like behavior were normalized. These findings indicate, that neurotransmission in the brain can be significantly impaired by the absence of normal gut microbiota and that this altered neurochemical profile persists despite the restoration of a normal microbiome later in life (Clarke et al., 2013).

## 10 Conclusion and future perspectives

Over the past decade, scientists and clinicians have been actively searching for basic mechanistic insight into the pathogenesis of highly prevalent mental disorders, including schizophrenia, depression, migraine, and neuropathic pain. Qualitative and quantitative changes in the gut microbiota and changes in the amount of neurotransmitters and metabolites of microbial origin have been identified, and support a relevant role of the gut-brain axis in the development of these conditions. Although the beneficial effects of probiotics, certain metabolites, and FMT on migraine and other neurological disorders favor this concept, there are major gaps in understanding their etiology and pathogenesis, and it is not yet clear which of the microbiome related neurotransmitters, metabolites, and pathways are causally involved. Nonetheless, the gut microbiome has an important influence on brain functions and mental health including pain disorders, and there is increasing interest in microbiota with probiotic properties, as novel and safe treatment options.

New technologies such as the use of reporter mouse lines and optogenetic tools have become available recently to specifically dissect the precise communication routes between the gut and the brain including the roles of particular cell types and neuron populations in the gut and the brain (Crock et al., 2012; Makadia et al., 2018; Spencer et al., 2018). Based on the emerging importance of the gut microbiome for the function of the entire organism and the bidirectional communications paths between the gut microbiome and the nervous system affecting mental health, novel opportunities for clinical applications of gut microbiome related therapies are expected to emerge for highly prevalent medical conditions including irritable bowel disease, migraine, and mental disorders.

## Author contributions

LR: Writing – review & editing. JF: Writing – review & editing. MK: Writing – review & editing. NR: Writing – review & editing.

## Figures and Tables

**Table:** 

AD	Alzheimer’s disease
ADHD	Attention-deficit-hyperactive disorder
AEA	Cannabinoid anandamide
AHLs	N-acyl-homoserine lactones
APC	Antigen presenting cell
ASD	Autism spectrum disorder
BBB	Blood brain barrier
BDNF	Brain derived neurotrophic factor
CC	Colonocytes
CGRP	Calcitonin gene-related peptide
CLRs	C-type lectin receptors
CNS	Central nervous system
DA	Dopamine
DRG	Dorsal root ganglia
EC	Enterocyte in the Glossary
FMT	Fecal microbiota transplantation
GC	Goblet cell
GF	Germ-free
GI	Gastrointestinal
5-HIAA	5-Hydroxy indole acetic acid
5-HT	Serotonin
IAAld	Indole-3-acetaldehyde
IBD	Inflammatory bowel disease
IEC	Intestinal epithelial cell
IEt	Indole-3-ethanol
ILA	Indole-3-lactic acid
IL	Interleukin
LPS	Lipopolysaccharides
KYN	Kynurenine
KYNA	Kynurenic acid
MAMPs	Microbe- associated molecular patterns
MDD	Major depressive disorder
MC	Microfold cell
NLRs	NOD-like receptors
NOD	Nucleotide-binding, and oligomerization domain
NTS	Nucleus tractus solitarii
PAMPs	Pathogen-associated molecular patterns
PANs	Primary afferent neurons
PC	Paneth cell
PD	Parkinson’s disease
PRRs	Pattern recognition receptors
QSM	Quorum sensing molecules
RIG-1	Retinoic acid inducible gene 1
RLRs	RIG-1-like receptors
SCFAs	Short chain fatty acids;
SZ	Schizophrenia
TBI	Traumatic brain injury
TC	Tuft cell
TL	Thoracolumbar
TLRs	Toll-like receptors
TNF-α	Tumor necrosis factor- α
TRP	Transient receptor potential
XA	Xanthurenic acid
